# Molecular Basis of the Inflammation Related to Obesity

**DOI:** 10.1155/2019/5250816

**Published:** 2019-02-17

**Authors:** Ana B. Crujeiras, Paul Cordero, Diego F. Garcia-Diaz, Ewa Stachowska, Pedro González-Muniesa

**Affiliations:** ^1^Epigenomics in Endocrinology and Nutrition Group, Epigenomics Unit, Instituto de Investigacion Sanitaria de Santiago (IDIS), Complejo Hospitalario Universitario de Santiago (CHUS/SERGAS), Santiago de Compostela, Spain; ^2^CIBERobn Physiopathology of Obesity and Nutrition, Centre of Biomedical Research Network, ISCIII, Madrid, Spain; ^3^Institute for Liver and Digestive Health, University College London, Royal Free Hospital, Rowland Hill Street, London, UK; ^4^Laboratorio de Nutrigenomica, Departamento de Nutricion, Facultad de Medicina, Universidad de Chile, Chile; ^5^Department of Biochemistry and Human Nutrition, Pomeranian Medical University, Szczecin, Poland; ^6^University of Navarra, Department of Nutrition, Food Science and Physiology, School of Pharmacy and Nutrition, Pamplona, Spain; ^7^University of Navarra, Centre for Nutrition Research, School of Pharmacy and Nutrition, Pamplona, Spain; ^8^IDISNA, Navarra's Health Research Institute, Pamplona, Spain

Almost two thousand millions of adults suffer from overweight or obesity in the world [1]. After decades of research, we understand that the solution to this problem is not easy, as the pernicious trend is still increasing [2].

This disease is defined as an excessive fat accumulation together with a moderate but chronic inflammation [3]. This accompanying proinflammatory status is considered the link between obesity and the development of its related comorbidities such as insulin resistance and type 2 diabetes, cardiovascular diseases, cancer, and nonalcoholic fatty liver disease [4].

The main triggers for this inflammation have been traditionally considered oxygen tension, oxidative stress, and endoplasmic reticulum stress [5]. This special issue contains articles analyzing oxidative stress, adipokine secretory pattern, methylation, or nanomedicine to further untangle the processes involved in this disease and to offer promising/possible alternative therapeutical tools.

In this sense, D. Stygar and collaborators measured in rats the levels of selected adipokines and other proteins, such as FABP4, leptin, chemerin, and CRP, to show the proinflammatory effect of a high-fat diet. Interestingly, this effect was reversed by the treatment of obesity with metabolic surgery. As mentioned earlier, it seems clear the relation of an abnormally enlarged adipose tissue and oxidative stress as it has been analyzed in the article of Y. Gramlich and coworkers. These authors suggest that obese patients undergoing coronary artery bypass grafting showed altered myocardial redox patterns, indicating an increased oxidative stress with inadequate antioxidant compensation. This might explain why patients with high BMI suffering from coronary artery disease are more susceptible to cardiomyopathy and possible damage by ischemia and reperfusion, for example, during cardiac surgery. On the other hand, oxidative stress seems to be related with diabetic retinopathy, one of the multiple accompanying side-effects of obesity [6]. In this sense, A. Maugeri and collaborators stated that hyperglycemia increased ROS production and alters the DNA methylation process, therefore altering the expression of certain genes. They also found in their study that curcumin seems to reduce oxidative stress and improves methylation activities, considering this compound an effective antioxidant to counteract this condition. In the same area, another potent antioxidant with self-regenerative properties, nanoparticles of cerium oxide, was tested by A. Lopez-Pascual and coworkers in three types of cells (adipocytes, macrophages, and myotubes) under proinflammatory conditions. These nanoparticles showed a mild insulin-sensitizing effect on murine adipocytes and myotubes that needs to be further studied. Finally, in another manuscript, M. Carreira et al. stated that *Sirt6* expression could be a potential therapeutic target to counteract obesity-related liver diseases because it is downregulated by the chronic low-grade inflammation and oxidative stress induced by excess adiposity.

In conclusion, it is recognized worldwide that the main predisposing factors to develop obesity are overconsumption of calories and sedentary lifestyle, although the reality is quite more complex [7]. These two options either together or individually will lead to an abnormal enlarged adipose tissue, accompanied by a proinflammatory status [8]. We have known this for many years but without being able to tackle the epidemy of obesity. Therefore, we need to understand the molecular pathways involved in this pathological condition to provide new therapeutic tools, being the reason for this special issue.
